# The American Institute of Homœopathy and the International Homœopathic Congress

**Published:** 1891-03

**Authors:** Pemberton Dudley

**Affiliations:** General Secretary, A. I. H.; S. W. Cor. 15th and Master Sts., Philadelphia, Pa.


### THE AMERICAN INSTITUTE OF HOMŒOPATHY AND THE INTERNATIONAL HOMŒOPATHIC CONGRESS.

SECRETARY'S NOTICE.

EDITORS OF THE HOMŒOPATHIC PHYSICIAN:—The American Institute of Homœopathy will hold its forty-fourth annual session and celebrate its forty-eighth anniversary in conjunction with the Fourth Quinquennial International Homœopathic Congress, at Atlantic City, New Jersey, beginning on Tuesday morning, June 16th, 1891. In accordance with action taken at its last session, the Institute will transact, as far as possible, its necessary routine business on that day, and the International Congress will assemble on the following morning. The sessions of the latter will occupy the morning and afternoon of each day (Sunday excepted) until Tuesday, June 23d. This arrangement of the business of the Institute makes it necessary that all the standing and special committees should have their reports in readiness before the opening of the session. But it should be noticed that all *scientific* reports of committees and bureaus appointed last year will be deferred until the session of 1892, thus giving place to the scientific work of the Congress. All members of homœopathic Medical Societies will have equal rights as members of the Congress, and equal privileges in the transaction of its business and in the discussions, under such rules as may be adopted for the government thereof. The *Transactions* will be published by the American Institute of Homœopathy and furnished to physicians on such terms as may be decided by the Executive Committee.

It is expected that the proceedings of the Congress will be of the most interesting and important character. While General Medicine, Surgery, Obstetrics, and the Specialties will have their place in the discussions, the interests of Homœopathy will furnish the main topics for consideration. It is proposed that one entire day—"Materia Medica Day"—shall be devoted to the consideration of the questions pertaining to its present status and its further improvement. Homœopathic Therapeutics will also claim a large share of attention, while some of the subjects upon which the homœopathic school is known to hold a distinctive position, will be presented and considered. The Essays and Addresses on all of these subjects will be presented by physicians carefully chosen by the committee having the matter in charge, and the discussions will be participated in by some of the physicians most distinguished in each department. Arrangements are in progress to secure reports of condition and advancement of Homœopathy in all the countries of the civilized world.

A word as to the place of meeting. Atlantic City, as is well known, extends for a distance of two or three miles along the sea-coast of New Jersey, sixty miles southeast of Philadelphia, with which it communicates by three lines of railway and scores of trains daily, most of which make the distance in ninety minutes. New York and Baltimore are within four or five hours' ride, while within a radius of four hundred miles there are nearly four thousand homœopathic physicians. Atlantic City has, during "the season," a larger patronage than any other of our sea-coast resorts, her visitors coming from all quarters of the country, but chiefly from New York, Philadelphia, Baltimore, and the West and South. She has ample hotel accommodations for twenty-five thousand guests. The United States Hotel, which will be the headquarters of the Congress and the place of its meetings, is a new structure, located one square from the beach and within full view of the ocean. It has accommodations for eight hundred guests, and the "pavilion" in which the Congress will assemble is a large room on the first floor, with a seating capacity for eight hundred persons. The meeting of the Congress will occur during "the season," but the United States Hotel will be practically at our exclusive disposal. The scientific and social features of the meeting and the attractions of Atlantic City as a health and pleasure resort render it probable that this Congress will be by far the largest gathering of homœopathic physicians ever convened. It is especially suggested that the occasion will furnish an unusual opportunity for our physicians to combine the profit of a scientific convention with the pleasures and benefits of a vacation, both for themselves and their families.

PEMBERTON DUDLEY, M. D.,
*General Secretary, A. I. H.*

S. W. COR. 15TH AND MASTER STS.,
PHILADELPHIA, PA.

